# Isolation, identification, and whole genome sequence analysis of the alginate-degrading bacterium *Cobetia* sp*.* cqz5-12

**DOI:** 10.1038/s41598-020-67921-7

**Published:** 2020-07-02

**Authors:** Wenwen Cheng, Xuanyu Yan, Jiali Xiao, Yunyun Chen, Minghui Chen, Jiayi Jin, Yu Bai, Qi Wang, Zhiyong Liao, Qiongzhen Chen

**Affiliations:** College of Life and Environmental Science, Wenzhou University, Wenzhou, 325035 People’s Republic of China

**Keywords:** Bacterial genomics, Marine microbiology, Genomic analysis, Isolation, separation and purification

## Abstract

Alginate-degrading bacteria or alginate lyases can be used to oligomerize alginate. In this study, an alginate-degrading bacterium with high alginolytic activity was successfully screened by using *Sargassum fusiforme sludge*. When the strain was grown on a plate containing sodium alginate, the transparent ring diameter (D) was 2.2 cm and the ratio (D/d) of transparent ring diameter to colony diameter (d) was 8.8. After 36 h in culture at a temperature of 28 °C shaken at 150 r/min, the enzymatic activity of the fermentation supernatant reached 160 U/mL, and the enzymatic activity of the bacterial precipitate harvested was 2,645 U/mL. The strain was named *Cobetia* sp. cqz5-12. Its genome is circular in shape, 4,209,007 bp in size, with a 62.36% GC content. It contains 3,498 predicted coding genes, 72 tRNA genes, and 21 rRNA genes. The functional annotations for the coding genes demonstrated that there were 181 coding genes in the genome related to carbohydrate transport and metabolism and 699 coding genes with unknown functions. Three putative coding genes, *alg2107, alg2108* and *alg2112,* related to alginate degradation were identified by analyzing the carbohydrate active enzyme (CAZy) database. Moreover, proteins Alg2107 and Alg2112 were successfully expressed and exhibited alginate lyase activity.

## Introduction

Alginate is a kind of water-soluble acidic polysaccharide found on the cell walls of algae, such as *Gigantophtha*, *Charcot algae*, *Laminaria japonica*, and *Sargassum fusiformis*. It is formed by the random combination of *beta*-d-1,4-mannosuronic acid and *alpha*-l-1,4-gulosuronic acid^1, 2^. Because of its high degree of polymerization, high molecular weight, and high viscosity, it not easily absorbed and utilized by the human body, which limits its potential applications^3^. The oligomerization of alginate can widen the scope of its potential applications and results in more biological activities. The oligomeric alginate is called alginate oligosaccharide (AOS). AOS, a kind of functional oligosaccharide, exhibits a variety of properties such as increasing fruit quality^4^, and antioxidative^5^, anticoagulant^6^, and antineoplastic^7^ potential. Moreover, AOS has broad application prospects in the fields of pharmaceuticals, functional food, and green agriculture. Nowadays, there are many ways to achieve alginate oligomerization. At present, it is recognized that the environmentally friendly and mildly efficient method of alginate oligomerization is to use alginate-degrading microorganisms or alginate lyase^8^ extracted from microorganisms. Until now, scientists have isolated several strains of alginate-degrading microorganisms, including *Cobetia* sp. NAP1^9^, *Microbulbifer mangrovi* DD-13^T^^10^, *Vibrio weizhoudaoensis* M010^11^, *Isoptericola halotolerans* NJ-05^12^, *V. splendidus* 12B01^13^, *Sphingomonas *sp. A1^14^, *Pseudoalteromonas* sp. CY24^15^, etc. Some alginate lyase enzymes have also been isolated and purified from these strains^11–13, 15^.

With the improvement of sequencing technology and the dramatic reduction in sequencing costs, the basic information about a target strain can be quickly obtained by using high-throughput technology to determine its whole genome sequence. The sequence of relevant coding genes can also be quickly obtained by means of gene annotation. Tang et al. obtained the complete genomic sequence information of *Cobetia marina* JCM 21022^T^ using high-throughput sequencing and determined the taxonomic status and functional differences between this strain and other strains of related families and genera by comparative genomics^16^. Md. Imran et al. also obtained the complete genomic information of *Microbulbifer mangrovi* DD-13^T^ using high-throughput sequencing, and identified a series of genes related to polysaccharide degradation through functional annotation^12^. Thereafter, the polysaccharide substrate spectrum of the strain was verified based on genetic information. By considering both the crude enzyme experiments and the whole genome sequence information, researchers determined that sodium alginate can be thoroughly degraded by *Algibacter alginolytica* sp. novHZ22, and proposed that the structure and mechanism of polysaccharide utilization loci (PUL) be the goal of further research^17^.

In order to select the most efficient alginate-degrading microorganism or alginate lyase for use in the industrial production of AOS, we needed to modify the present alginate-degrading microbe or alginate lyase, or else isolate a new, more efficient alginate-degrading microorganism, and purify a new alginate lyase from it. For this purpose, the strain *Cobetia* sp. cqz5-12 was isolated from *Sargassum fusiformis* sludge taken from the Dongtou sea area, Wenzhou, Zhejiang Province, China. In order to obtain basic information about the strain quickly, whole genome sequencing of the strain was performed by using the Illumina Miseq + Pacbio sequencing platform. Through bioinformatics analysis, the complete genomic information about the strain was obtained, from which the coding gene sequences potentially related to alginate degradation were obtained and attempts were made to express these sequences. The identification, genome sequencing, and target gene expression of *Cobetia* sp. cqz5-12 aid in the better understanding of the specific alginolytic properties of this strain. Further research can be performed using this data with the goals of understanding the degrading mechanism at the molecular level and, in turn, improving its alginolytic efficiency.

## Results

### Isolation and identification of alginate-degrading strains

**Figure 1:**
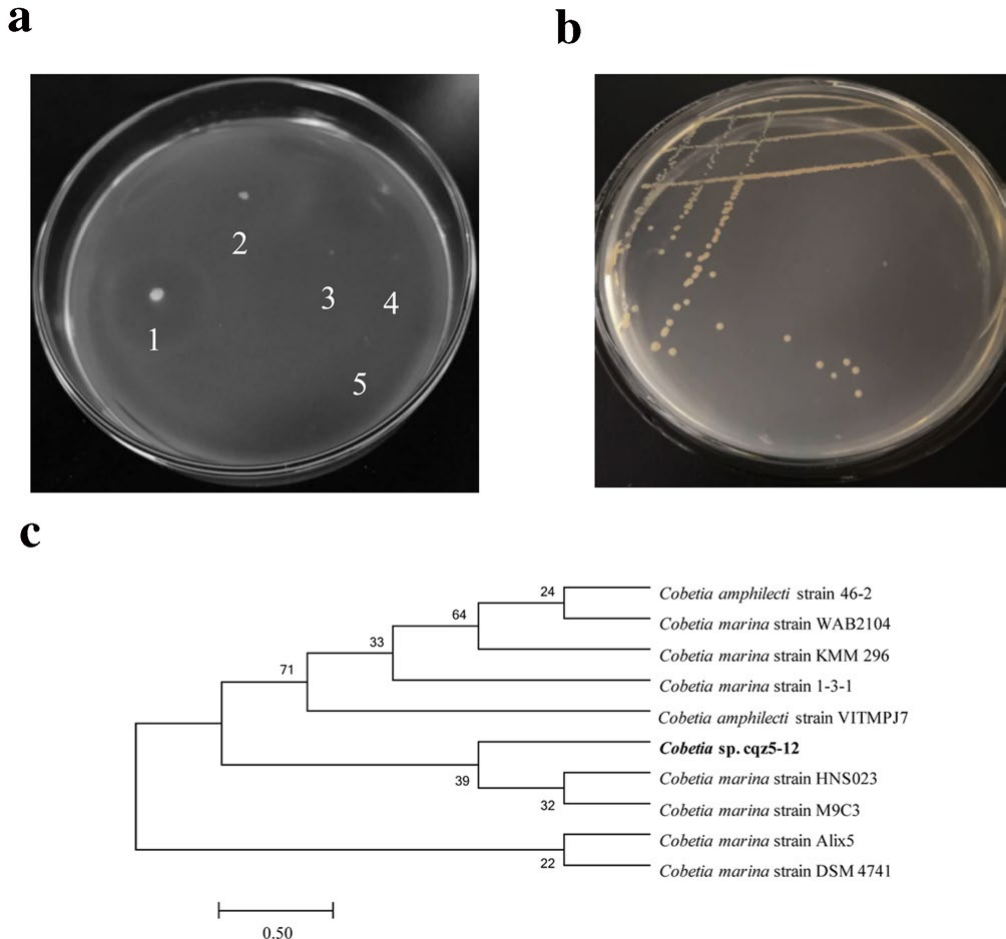
Degradation effect of alginate-degrading bacterium *Cobetia* sp. cqz5-12 on a plate containing sodium alginate and identification of the strain. (**a**) The results of plate degradation of alginate-degrading bacteria. 1: *Cobetia* sp. cqz5-12 with degradation effect, 2–5: the strains that lack the ability to degrade sodium alginate. (**b**) The colony morphology of *Cobetia* sp. cqz5-12 on solid medium A containing 3% NaCl. The photographs in (**a**) and (**b**) were captured by co-author Wenwen Cheng using camera (Sony, DSC-P51, USA) at laboratory in WenZhou University. (**c**) The construction of a phylogenetic tree using MEGA 7.0 software. The analysis of the self-expansion was performed after 1,000 times of repeated sampling by the Neighbor-joining method^53^.

In this research, a strain of bacterium capable of degrading alginate was successfully isolated from rotten *Sargassum fusiformis*. When a colony of this strain formed on a solid plate containing sodium alginate, 1% calcium chloride was used to soak the colony for 30 min, and then a transparent circle could be clearly observed around the colony (Fig. 1a). The diameter of the colony (d) was 0.25 cm, the diameter of the transparent ring (D) was 2.2 cm, and the D/d value (the ratio value of transparent ring diameter to colony diameter) was 8.8. The strain was then inoculated into a liquid fermentation medium containing 0.6% sodium alginate. After 36 h at 28 °C and 150 r/min, the enzyme activity of the supernatant was 160 U/mL. The precipitate of bacteria was lysed by ultrasonication, then the enzymatic activity of the supernatant was measured, which reached up to 2,645 U/mL The significant difference in enzymatic activity between the fermentation supernatant and the cell disruption supernatant revealed that the alginate lyase produced by the bacteria was mostly retained inside the cell, while some was secreted to the extracellular space and played a role in the observed enzymatic activity.

After incubation on solid medium A containing 3% NaCl at 28 °C for 48 h, the colony of this alginate-degrading strain was pale yellow, round (diameter 1–1.2 mm), moist, and smooth (Fig. 1b). Gram staining and microscopic observation showed that the alginate-degrading strain was a short gram-negative rod (0.1–0.2 μm × 0.3–0.4 μm). Blast analysis of 16S rDNA sequence showed that the similarity of the 16S rDNA sequence of the strain was as high as 99% with that of the strain *Cobetia*. Using MEGA 7.0 for cluster analysis, we found that the strain could be clustered with the strain *Cobetia marine* (Fig. 1c). Therefore, the strain was named *Cobetia* sp*.* cqz5-12.

### Basic information about the genome of the alginate-degrading strain

**Figure 2:**
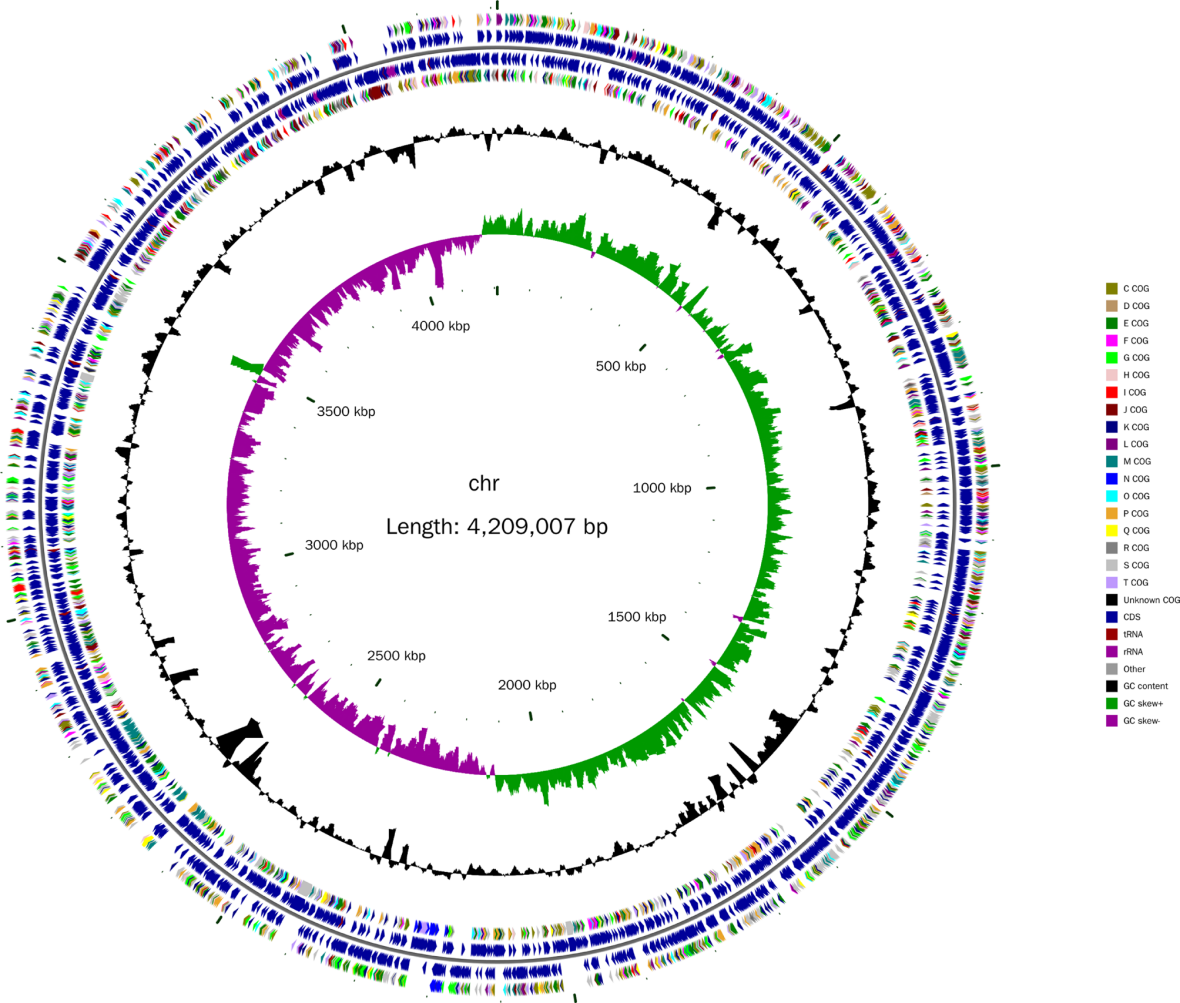
Graphical circular map of the *Cobetia* sp. cqz5-12 chromosome. From the inside to the outside, the first circle represents the scale; the second circle represents the GC Skew; the third circle represents the GC content; the fourth and seventh circles represent the COG to which each CDS belongs; the fifth and sixth circles represent the position of CDS, tRNA, and rRNA in the genome. COG classification is based on the KEGG pathway database^50–52^.

Basic information about the whole genome sequence of *Cobetia* sp. cqz5-12 was obtained using high-throughput sequencing (Fig. 2). The genome of the strain is circular, 4,209,007 bp in size with 62.36% GC content (Table 1). GeneMarkS (version 4.32 April 2015) software was used to predict the whole genome sequence^18^ and 3,498 coding sequences (CDS) were obtained. The total length of the CDS was 3,525,474 bp, accounting for 83.76% of the total genome length. The average length of the CDS was 1,007.85 bp, the average GC content of the CDS was 63.50%, and the maximum CDS length was 20,397 bp. By predicting non-coding RNA, 21 rRNAs (5s + 16s + 23s), 72 tRNAs, and 114 other ncRNAs were obtained, accounting for 0.7534%, 0.1337% and 0.7522% of the total length of the genome sequence, respectively (Table 1). The predictive analysis of functional elements and subsystems of the genome revealed that the genome had no plasmid and CRISPRs (Clustered Regularly Interspaced Short Palindromic Repeats) structure, but had a complete prophage, 20 antibiotic resistance genes (0.0057% of the total CDS), 20 antibiotic target genes (0.0057% of total CDS), and one antibiotic biosynthesis-related gene (0.0003% of total CDS).

**Table 1:** Genome assembly and general features of *Cobetia* sp. cqz5-12 genome.

Category	Number	Percentage of genome (%)
Total number of bases (assembly size)	4,209,007	100
DNA G + C number of bases	2,624,737	62.36
CDS number of bases (bp)	3,525,474	83.76
Total number of CDS	3,498	83.76
Genes in COGs	3,126	74.86
Total number of RNA genes	207	1.6393
rRNA genes	21	0.7534
5S rRNA	7	0.0185
16S rRNA	7	0.2555
23S rRNA	7	0.4794
tRNA genes	72	0.1337
other ncRNA	114	0.7522

### Subcellular localization and functional annotation analysis of the CDS

Subcellular localization analysis of 3,498 coding sequences (CDS) revealed that 236 protein-coding genes contained signal peptide sequences, accounting for 6.75% of the total CDS, and there was at least one transmembrane helical region in 872 CDS, accounting for 24.9% of the total CDS. There were sequences of secretory proteins in 78 coding genes, accounting for 4.95% of the total CDS.

Sequence blast of the CDS and functional annotation analysis of CDS were performed. The analysis results showed that 229 CDS were related to energy production and conversion, 181 were related to carbohydrate transport and metabolism, 169 were related to translation, ribosomal structure and biogenesis, 224 were related to transcription, 200 were related to cell wall/membrane/envelope biogenesis, 29 were associated with cell motility, 230 were associated with inorganic transport and metabolism, 58 were associated with secondary metabolites biosynthesis, transport and catabolism, 138 were associated with signal transduction mechanisms, 57 were related to intracellular trafficking, secretion and vesicular transport, and the function of 699 CDS were unknown (Table 2). One CDS of unknown function has been reported to encode a completely new protein^19^. Therefore, about 20% of the CDS of this strain may encode new proteins.

**Table 2:** Clusters of Orthologous Groups of proteins functional categories of genes from *Cobetia* sp. cqz5-12.

COG categories	Categories function	ORF number	Percentage (%)^a^
A	RNA processing and modification	1	0.0286
C	Energy production and conversion	229	6.5466
D	Cell cycle control, cell division, chromosome partitioning	31	0.8862
E	Amino acid transport and metabolism	312	8.9194
F	Nucleotide transport and metabolism	77	2.2013
G	Carbohydrate transport and metabolism	181	5.1744
H	Coenzyme transport and metabolism	122	3.4877
I	Lipid transport and metabolism	88	2.5157
J	Translation, ribosomal structure and biogenesis	169	4.8313
K	Transcription	224	6.4037
L	Replication, recombination and repair	118	3.3734
M	Cell wall/membrane/envelope biogenesis	200	5.7176
N	Cell motility	29	0.8290
O	Posttranslational modification, protein turnover, chaperones	127	3.6306
P	Inorganic ion transport and metabolism	230	6.5752
Q	Secondary metabolites biosynthesis, transport and catabolism	58	1.6581
S	Function unknown	699	19.9828
T	Signal transduction mechanisms	138	3.9451
U	Intracellular trafficking, secretion, and vesicular transport	57	1.6295
V	Defense mechanisms	36	1.0292

^a^Percentage has been calculated based on total number of CDS (3,498).

### Expression and purification of polysaccharide lyases Alg2107, Alg2108 and Alg2112

The analysis of carbohydrate-active enzymes (CAZy) of the CDS was performed by using the CAZy database and hmmscan software (3.1b2, February 2015)^20, 21^. The database mainly contains enzyme families related to the degradation, modification, and generation of glycoside bonds. The CAZy analysis showed that *Cobetia* sp. cqz5-12 contained four polysaccharide lyases (PLs), 17 glycoside hydrolases (GHs), 39 glycosyltransferases (GTs), 14 carbohydrate esterases (CEs), seven carbohydrate binding modules (CBMs), and 11 Auxiliary Activities (AAs).

Based on the result of CAZy analysis, the four polysaccharide lyases are encoded by genes numbered 1771, 2107, 2108 and 2112. The genes were named *alg1771*, *alg2107*, *alg2108*, and *alg2112*, respectively. *alg1771*, 1,272 bp in length, was annotated as the transporter TolB; *alg2107, alg2108* and *alg2112*, the lengths of which were 2,142, 2,160 and 915 bp, respectively, were annotated as hypothetical proteins, oligosaccharide lyases and polysaccharide lyases, respectively. Furthermore, the enzymatic activities of the fermentation supernatant and the cell disruption supernatant of *Cobetia* sp. cqz5-12 were 160 and 2,645 U/mL respectively, which indicated that the alginate lyase produced by the bacteria was mostly retained inside the cell. According to this lyase assay result, we thought that *alg1771* might play a minor role in the secretion of alginate lyase. Based on these data, *alg2107, alg2108,* and *alg2112* were selected for further experimental verification.

**Figure 3:**
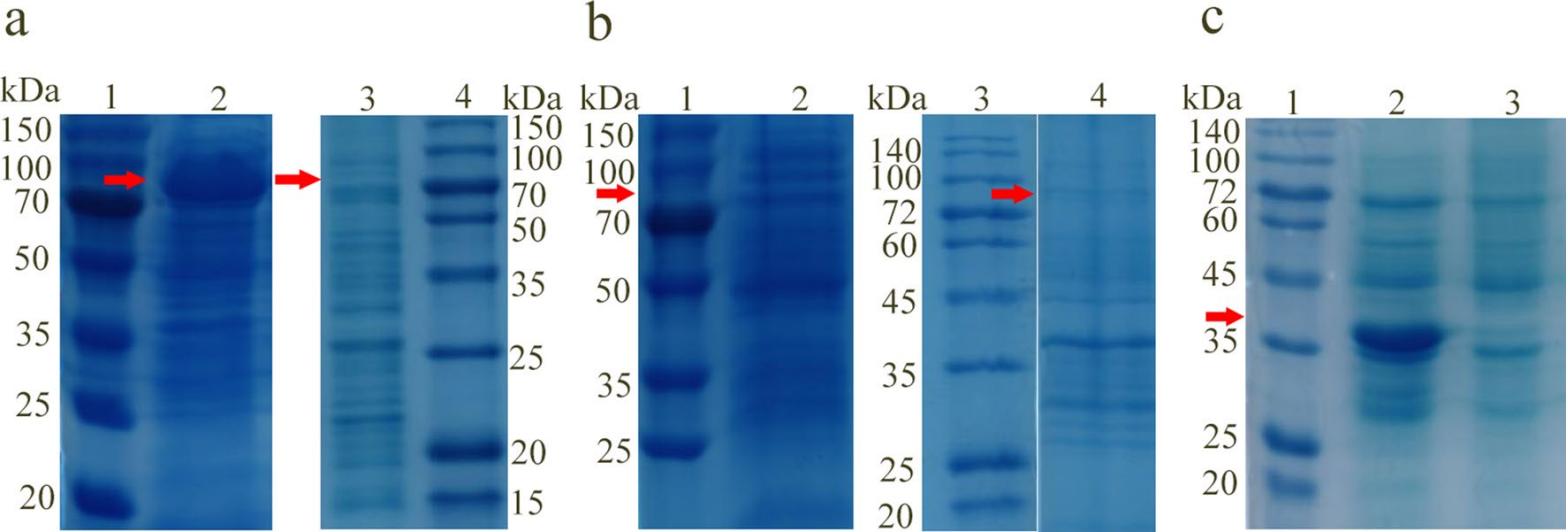
The SDS-PAGE analysis of crude extracts of *E. coli* BL-07, *E. coli* BL-08 and *E. coli* BL-12 that expressed Alg2107, Alg2108, and Alg2112, respectively. (**a**) The electrophoretic results from crude extract of *E. coli* BL-07 that expressed Alg2107. Lane 1 is the Protein Marker, Lane 2 is the crude extract of *E. coli* BL-07 that expressed Alg2107, Lane 3 is the protein crude extract of the negative control expressing empty pET-29a plasmids, Lane 4 is the Protein Marker. The red arrow indicates the theoretically predicted position of protein Alg2107, the samples derive from the same experiment and gels were processed in parallel. The full-length gels are presented in Supplementary Figure S1; (**b**) the electrophoretic results from crude extract of *E. coli* BL-08 that expressed Alg2108. Lane 1 is the Protein Marker, Lane 2 is the crude extract of *E. coli* BL-08 that expressed Alg2108, Lane 3 is the Protein Marker, Lane 4 is the protein crude extract of the negative control expressing empty pET-29a plasmids. The red arrow indicates the theoretically predicted position of protein Alg2108, the samples derive from the same experiment and gels were processed in parallel. The full-length gels are presented in Supplementary Figure S2; (**c**) the electrophoretic results from crude extract of *E. coli* BL-12 that expressed Alg2112. Lane 1 is the Protein Marker, Lane 2 is the crude extract of *E. coli* BL-12 that expressed Alg2112, Lane 3 is the protein crude extract of the negative control expressing empty pET-29a plasmids. The red arrow indicates the theoretically predicted position of protein Alg2112, the samples derive from the same experiment and gels were processed in parallel. The full-length gels are presented in Supplementary Figure S3.

The SDS-PAGE analysis of the crude protein extract showed that Alg2107 and Alg2112 successfully appeared in the theoretically expected position, while Alg2108 did not (Fig. 3). The theoretical band sizes of Alg2107, Alg2108, and Alg2112 are 80.19, 80.64, and 35.4 KDa, respectively. In addition, Alg2108 was not detected by Western blotting. These results indicated that Alg2107 and Alg2112 were expressed successfully, but that Alg2108 was not. The selection of host strain, expression vector, and culture environment will affect the final expression effect of a protein. Therefore, further exploration of the optimal conditions for the successful expression of Alg2108 is necessary. Using sodium alginate as substrate, the degradation activity of the three crude protein extracts was detected using the DNS method, and the results showed that the activity of Alg2107, Alg2108, and Alg2112 crude extracts was 686, 0, and 486 U/mL, respectively. The specific activities of these crude extracts were 142, 0, and 129 U/mg, respectively. After purification, the specific activities of purified Alg2107, Alg2108, and Alg2112 were 260, 0, and 281 U/mg, respectively. The enzyme activity tests showed that Alg2107 and Alg2112 exhibit alginate lyase activity, while Alg2108 did not degrade sodium alginate.

## Discussion

Alginate is the main component on the cell wall of brown algae. At present, scientists have isolated and purified a large number of alginate-degrading marine microorganisms and alginate lyases from decaying algae, seawater, or seawater sediment, such as *Vibrio* sp*.* QD-5 isolated from rotting kelp^22^ and alginate lysase AlgA from the marine bacterium *Bacillus* sp. Alg07^23^. *Cobetia* sp. cqz5-12, the research topic of this study, was isolated from rotten *Sargassum fusiformis*. After incubation in a shaking culture containing sodium alginate, the sodium alginate lyase activity of the culture supernatant fluid was 160 U/mL, significantly lower than the value of 510 U/mL found in the marine bacterium *Bacillus* sp. Alg07^23^ reported previously. However, the enzymatic activity of cell disruption supernatant of *Cobetia* sp. cqz5-12 reached 2,645 U/mL. Nevertheless, the enzymatic activity of the cell disruption supernatant of *Bacillus* sp. Alg07 has not been reported. Both the culture supernatant of *Cobetia* sp. cqz5-12 and the cell disruption supernatant of *Cobetia* sp. cqz5-12 had alginolytic activity, indicating that there may be more than one enzyme in strain cqz5-12 capable of degrading alginate. Some of the reported alginate lyases are intracellular enzymes^22^, which can be obtained from alginate-degrading bacteria by ultrasonication^24^ or purification after heterologous expression^25^. Others are extracellular enzymes^23, 26–29^, which can be purified directly from the culture supernatant of the alginate-degrading bacteria, such as AlyV5^27^. Therefore, we believe that strain cqz5-12 may produce multiple enzymes, both intracellular and extracellular, which can degrade alginate.

Using high-throughput sequencing techniques, the complete genomic information of *Cobetia* sp. cqz5-12 was obtained. The genome size of *Cobetia* sp. cqz5-12 was 4,209,007 bp and its GC content was 62.36%. Compared with the most homologous strain, *Cobetia Marina* JCM 21022^T^^12^, the genome size and GC content of strain cqz5-12 were not significantly different from those of the strain JCM 21022^T^ (The genome size of *C. Marina* JCM 21022^T^ is 4,176,300 bp and its GC content is 62.44%). However, the gene involved in alginate degradation may differ between strains cqz5-12 and JCM 21022^T^ (see Supplementary Table S2 online). Strain cqz5-12 contained 3,498 predicted coding genes (CDS), lower than the number of CDS found in the alginate-degrading bacteria *C. marina* JCM 21022^T^ (3,611), close to 3,488^10^ CDS found in alginate-degrading bacterium *Microbulbifer mangrove* DD-13^T^, but higher than that of the alginate-degrading bacterium *Algibacter alginolytica* HZ22 (3,371)^17^. Alginate-degrading bacterium *Algibacter alginolytica* HZ22 and *Mirobulbifer mangrovi* DD-13^T^ contained 255 (18 PLs) and 461 CAZymes (17 PLs), respectively, while *Cobetia* sp. cqz5-12 only had 92 CAZymes (4 PLs), much fewer than those previously reported in other strains. Although strain cqz5-12 contained fewer CAZymes, two of the four PLs genes were annotated as enzymes associated with alginate lysis (Table 3). The genome of strain cqz5-12 contained 181 coding genes related to carbohydrate transport and metabolism, and 699 coding genes with unknown functions. All of this information suggests that strain cqz-12 still has a good potential for use in alginate degradation, which has been verified by previous plate tests and cell enzymatic activity experiments.

**Table 3:** General features of four predicted genes according to the result of CAZy analysis.

Gene	Length (bp)	Function description	CAZy family	Prediction of signal peptides	Prediction of transmembrane helix	Prediction of secretory protein
*alg1771*	1,272	Translocation protein TolB	PL22	–^a^	–	–
*alg2107*	2,142	Hypothetical protein	PL17	–	–	–
*alg2108*	2,160	Oligo alginate lyase	PL17	–	–	–
*alg2112*	915	Polysaccharide lyase family 7 protein	PL7	–	–	–

^a^Represent that the prediction result is negative.

The genes coding for the four polysaccharide lyases obtained by CAZy analysis were *alg1771, alg2107, alg2108,* and *alg2112*, belonging to the 22nd, 17th, 17th, and 7th families of polysaccharide lyase (PL), respectively (Table 3). At present, most of the reported alginate lyases belong to the 7th, 14th, 15th, and 17th families of the PL proteins. Considering both the annotation information of the four genes and the results of the previous enzymatic activity experiments, the genes *alg2107, alg2108,* and *alg2112* were selected for further expression experiments and study. It is worth noting that, among the three genes, the expression of *alg2108* did not take place. There are many factors affecting the expression of *alg2108*, such as induction temperature, IPTG concentration, selection of expression vectors, host selection, and so on. Therefore, the absence of *alg2108* expression may be attributed to multiple factors, and further investigation of optimal expression conditions for *alg2108* should be undertaken.

Using sodium alginate as the substrate, the specific activities of purified Alg2107, Alg2108, and Alg2112 were 260, 0, and 281 U/mg, respectively. The properties of these enzymes were compared with previously reported enzymes and it was found that the specific activities of purified Alg2107 and Alg2112 were not the highest or lowest ones (Table 4). However, the specific activities of these two enzymes could be the second highest ones in reaction conditions of pH value was 7.5–8.0 or reaction temperature of 30–35 °C. When the reaction pH value was 7.5–8.0, the most active protein was AlgA and its optimal temperature was 40 °C, while the optimal temperature for Alg2107 and Alg2112 activities was 30–35 °C. Furthermore, when the reaction temperature was 30–35 °C, the most active protein was Aly-IV and its optimal pH was 8.9, while the optimal pH of Alg2107 and Alg2112 was 7.5–8.0 (Table 4). These differences in reaction pH and temperature indicated that Alg2107 and Alg2112 have a greater potential advantage than many alginate lyases in industrial applications and health care. No lyase activity on the part of Alg2108 was detected in the enzyme activity assay, which may be related to a very low level of protein expression or inappropriate substrates used (Alg2108 was annotated as an oligosaccharide lyase, and the most suitable substrate was an oligosaccharide rather than a polysaccharide, such as sodium alginate). The enzymatic activities of Alg2107 and Alg2112 crude extracts were 686 and 486 U/mL, respectively, which were much lower than that of the cell disruption supernatant of strain cqz5-12 (2,645 U/mL). Alg2107 and Alg2112 are not secretory proteins as they do not contain a signal peptide (Table 3). Thus, they are intracellular enzymes. The lyases in the cell disruption supernatant of strain cqz5-12 are also intracellular enzymes. The significant difference in enzyme activity between the expressed protein crude extracts and strain cqz5-12 indicated that there were other proteins (aside from Alg2107 and Alg2112) produced by strain cqz5-12 that can degrade alginate. This deduction also confirms the hypothesis that alginate can be degraded by various enzymes (both intracellular and extracellular enzymes) produced by strain cqz5-12. In the process of heterologous expression, the excessive expression of a protein, or the absence and deficiency of cofactors, might lead to the lower enzymatic activity of the heterologous expressed protein observed compared with the protein generated from the original strain. In this study, the enzyme activities of heterologous Alg2107 and Alg2112 were significantly lower than those of the cell disruption supernatant of strain cqz5-12. This difference was possibly due to heterologous expression, but it was more likely due to the fact that Alg2107 and Alg2112 were only two of several alginate-degrading proteins produced, or, more likely, strain cqz5-12 employs multiple enzymes simultaneously to degrade alginate. Specific information about the intracellular and extracellular alginate-degrading proteins produced by strain cqz5-12 should be further investigated.

**Table 4:** Comparison between the properties of purified Alg2107 and Alg2112 and other previously reported enzymes.

Enzyme	Source	Specific activity (U/mg)	Molecular mass (kDa)	Optimal temperature (°C)	Optimal pH	Substrate specificity
Alg2107	This study	260	80.19	30	7.5	a
Alg2112	This study	281	35.4	35	8.0	a
Aly-IV	*Vibrio* sp. QD-5^22^	1,256.78	62	35	8.9	b
AlgA	*Bacillus* sp. Alg07^23^	8,306.7	60	40	7.5	c
Aly-W02	*Cobetia* sp. WG‐007^24^	21,285.5	35	45	8.5	b, c, alginate
OalS6	*Shewanella* sp. Kz7^25^	33.7	85.2	40	7.2	b, c
QY102	*Vibrio* sp. QY102^26^	254	28.5	40	7.1	b, c
AlySY08	*Vibrio* sp. SY08^27^	1,070.2	33	40	7.6	b
AlyH1	*Vibrio furnissii* H1^28^	2.40	35.8	40	7.5	b, c
ALW1	*Microbulbifer* sp. ALW1^29^	1.49	26	45	7.0	b, c
FsAlyPL6	*Flammeovirga* sp. NJ-04^30^	483.95	83.09	45	9.0	b, c, alginate
Aly1281	*Pseudoalteromonas carrageenovora* ASY5^31^	1.15	40.65	50	8.0	b, c
MSEA04	*Pseudomonas stutzeri* MSEA04^32^	116	40	37	7.5	alginate
QY105	*Vibrio* sp. QY105^33^	2,152	37	38	7.0	b, c
SjAly	*Saccharina japonica*^34^	13.8	35	30	8.0	b, c

^a^Represent no study.

^b^Abbreviation of poly α*-l*-guluronate (polyG).

^c^Abbreviation of poly β-d-mannuronate (polyM).

## Methods

### Reagents, medium, strains and plasmids

The rotten *Sargassum fusiformis* used in this study was collected from the sea area of the Dongtou District, Wenzhou City, Zhejiang Province.

The sodium alginate used in the medium was purchased from Sigma Company, and the remainder of the chemical reagents used was purchased from Sinopharm.

The formulation of the selective liquid medium used in the strain-isolating experiment was: 5 g/L sodium alginate, 1.0 g/L MgSO_4_, 5.0 g/L (NH4)_2_SO_4_, 30.0 g/L NaCl, 2.0 g/L K_2_HPO_4_, and 0.01 g/L FeSO4·7H_2_O at pH 7.0. The solid medium A was prepared by adding 1.8% agar powder to the above medium. The liquid fermentation medium was formulated as 6.0 g/L sodium alginate, 5.0 g/L tryptone, 2.5 g/L yeast powder, 25.0 g/L NaCl, 0.25 g/L KH_2_PO_4_, 0.5 g/LMgSO_4_·7H_2_O, and 0.05 g/L FeSO_4_·7H_2_O at pH 7.5. The formula of the seed liquid medium was the same as that of the liquid fermentation medium.

The other strains and plasmids used in the experiment, including *Escherichia coli* DH10B, *E. coli* BL21 (DE3) and pET29a, all came from our laboratory’s strain bank, except that the pMD 19-T vector was purchased from TaKaRa, and the degrading strain was isolated from rotting *Sargassum fusiformis*.

### Isolation and identification of degrading strains

1 g naturally rotted *Sargassum fusiforme* was cut into small pieces with sterile scissors, placed in 10 mL of selective liquid medium, and incubated in a shaking culture at 150 r/min and 28 °C. Twelve hours later, 1 mL of the bacterial culture solution was removed and added to 25 mL of fresh selective liquid culture medium. Incubation was continued with shaking at 150 r/min at 28 °C. After 48 h, 1 mL of the bacterial suspension was removed and diluted with sterile seawater adjusted to 10^–4^ gradient, then 100 μL of the dilution was applied to solid medium A. When a single colony developed, it was immediately transferred to a fresh solid medium A. After the colonies had grown out on the new plate, 1% calcium chloride solution was added and soaking took place for 30 min to observe whether transparent circles or white halos around the colonies were present. Single colonies within transparent circles or white halos were transferred to a new solid medium and incubation was continued. After the colonies grew out, a single colony was selected and inoculated into the seed liquid culture medium. Shaking culture proceeded overnight at 28 °C and 150 r/min. After harvesting the bacteria, the seed liquid was inoculated into 25 mL liquid fermentation medium, and cultured at 28 °C and 150 r/min for 36 h. The bacterial solution was centrifuged for 5 min at 4 °C and 12,000 r/min. The supernatant was removed and enzyme activity was determined. The strains with higher enzymatic activity were selected. The strains obtained were further screened by flask-shaking fermentation, and the strain with the highest alginate-degrading ability was finally selected.

Gram staining and microscopic observation was carried out on the alginate-degrading strains. Meanwhile, genomic DNA was extracted. The 16S universal primers 27F (5′-AGAGTTTGATCCTGGCTCAG-3′) and 1492R (5′-TACGGCTACCTTGTTACGACTT-3′) were used in the PCR reaction, and the products of the PCR reaction were purified, inserted into a pMD-19T vector, and then transformed into *E. coli* DH10B. The positive transformants were selected and sent to a biotechnology company for sequencing. Blast analysis was carried out on the sequencing results, and a phylogenetic tree was constructed using MEGA 7.0 software.

### Genome sequencing, assembly, and annotation

Genomic DNA was extracted according to the method proposed by Marmur and Doty^35^. The whole genome sequencing project was carried out by Shanghai Personal Biotechnology Co., Ltd. The whole genome shotgun strategy was used to construct the library of different inserts. The libraries were sequenced separately by using next-generation sequencing (Illumina MiSeq sequencing platform), as well as third-generation single-molecule sequencing (PacBio sequencing platform). The off-line data were obtained after DNA extraction, purification, library building, and sequencing. Further, the data obtained using second-generation sequencing were filtered by the filter standard to obtain high-quality data^36, 37^. The off-line data obtained from the third-generation sequencing were assembled using HGAP4 software^38, 39^, then the contig sequences were obtained based on these analyses. The contig sequences were adjusted by high-quality data via Pion (version 1.22) software^40^, and then the adjusted sequences were spliced to obtain the complete sequence.

The genes of the whole genome sequence were predicted by GeneMarkS (version 4.32 April 2015) software^18^. The predictions of tRNA genes, rRNA genes, and other non-coding RNAs genes were achieved by using tRNAscan-SE (version 1.3.1) software^41^, Barrnap (0.9-dev) (https://github.com/tseemann/barrnap), and the Rfam database, respectively^42^. Sequence blast of a protein coding sequence (CDS) was performed using diamond software (v0.9.10.111)^43^ and the NCBI nr database (release 2017.10.10). We also predicted signal peptide^44^, transmembrane helix^45^ and secretory protein encoding genes, and then performed the annotation of eggNOG (evolutionary genealogy of genes: Non-supervised Orthologous Groups)^46^, KEGG (Kyoto Encyclopedia of Genes and Genomes)^47^ and GO^48^. The CAZy (Carbohydrate-Active enZYmes Database) analysis^20, 21^ was also carried out. The whole genome map was drawn via cgview^49^, and Photoshop CS was used to edit the map.

### Cloning, expression, and purification of Alg2107, Alg2108, and Alg 2112

The gene fragments *alg2107, alg2108* and *alg2112* were obtained by PCR (for all PCR primers sequences see Supplementary Table S1 online) and inserted into the pMD19-T cloning vector. Thus, the recombinant cloning plasmids pMD19T-*alg2107*, pMD19T-*alg2108* and pMD19T-*alg2112* were obtained. The recombinant cloning plasmid pMD19T-*alg2107* was digested with Scal and HindIII restriction endonucleases, and the recombinant cloned plasmids pMD19T-*alg2108* and pMD19T-*alg2112* were digested with BamHI and SalI restriction endonucleases. Then, the target genes digested by the enzymes were ligated to the pET29a(+) vector and the recombinant expression vectors pET29a-Alg2107, pET29a-Alg2108, and pET29a-Alg2112 were obtained. Subsequently, the recombinant expression vectors were successfully transformed into the host strain *E. coli* BL21 (DE3) for protein expression, and the expression strains of *E. coli* BL-07, *E. coli* BL-08 and *E. coli* BL-12 were obtained.

A single colony of each protein expression strain was inoculated into LB (Luria–Bertani) liquid medium containing kanamycin and incubated overnight at 37 °C with shaking. The overnight culture was transferred to LB liquid medium containing kanamycin and cultured at 37 °C in a concussive manner.

When the OD_600_ value of the bacterial solution reached approximately 0.5, a final concentration of 0.2 mmol/L isopropyl *beta*-D-1-thiogalactopyran glycoside (IPTG) was added. Then the strains were cultured at 20 °C in a concussive manner for 24 h and harvested. The bacteria were precipitated by centrifugation at 8,000 r/min for 5 min. The bacterial precipitate was re-suspended in buffer A (10 mmol/L imidazole, 500 mmol/L NaCl and 20 mmol/L Tris–HCl; pH 7.0) and fragmented by ultrasound for 2 min. After 20 min of centrifugation at 4 °C and 12,000 r/min, the cell disruption supernatant was collected and used to for enzyme activity determination and further protein purification. The cell disruption supernatant was then purified through Ni–NTA chromatography, dialysis, and ultrafiltration.

Protein concentrations were determined by Bradford method and protein electrophoresis was performed using the SDS-PAGE method.

### Enzyme activity determination

The 3,5-dinitrosalicylic acid (DNS) method was used to detect the amount of reducing sugar released by enzymatic reaction. Then, the enzymatic activities of the alginate-degrading proteins were calculated according to the increase in reducing sugar content. A suspension of 0.6% of the sodium alginate substrate solution was prepared in disodium hydrogen Phosphate-citric acid buffer at pH 7.4. 1 mL properly diluted crude extract or purified enzyme solution was mixed with 1 mL of the substrate solution in a colorimetric tube, and then incubated in a water bath at 40 °C for 20 min. After adding 1.5 mL DNS solutions, the sample was immersed in a boiling water bath for 5 min, then immediately cooled to room temperature. The absorbance at 540 nm was determined after setting the sample volume to 10 mL. In blank controls, 1 mL inactivated crude extract was added to the substrate.

A unit of enzyme activity was defined as the amount of enzyme required to catalyze substrate to produce 1 μg of reducing sugar per minute. All enzymatic reactions were performed in three parallel experiments.

### Login number of the gene sequence

The complete genome sequence of *Cobetia* sp. cqz5-12 has been deposited at the DDBJ/EMBL/GenBank database under accession number CP044522.

### Collection number of *Cobetia* sp. cqz5-12

*Cobetia* sp. cqz5-12 has been deposited in the China General Microbiological Culture Collection Center, and the collection number is CGMCC 1.17459.

## Supplementary information

Supplementary file1 (PDF 676 kb)
