# Supplementary material for: Isolation, identification, and whole genome sequence analysis of the alginate-degrading bacterium Cobetia sp. cqz5-12
Source: Sci Rep. 2020 Jul 2;10:10920. doi: 10.1038/s41598-020-67921-7 (PMC7331586; doi:10.1038/s41598-020-67921-7)
Supplement: Supplementary file 1 — Supplementary file1 (PDF 676 kb) [file 41598_2020_67921_MOESM1_ESM.pdf]

Supplementary Information

Isolation, Identification, and Whole Genome Sequence Analysis of the Alginate-Degrading Bacterium *Cobetia* sp. cqz5-12

Wenwen Cheng[1], Xuanyu Yan[1], Jiali Xiao[1], Yunyun Chen[1], Minghui Chen[1], Jiayi Jin[1], Yu Bai[1], Qi Wang[1], Zhiyong Liao[1*], Qiongzhen Chen[1*]

1 College of Life and Environmental Science, Wenzhou University, Wenzhou, People's Republic of China, 325035

* Corresponding author: Zhiyong Liao, 1339456286@qq.com; Qiongzhen Chen, ashelychan@126.com

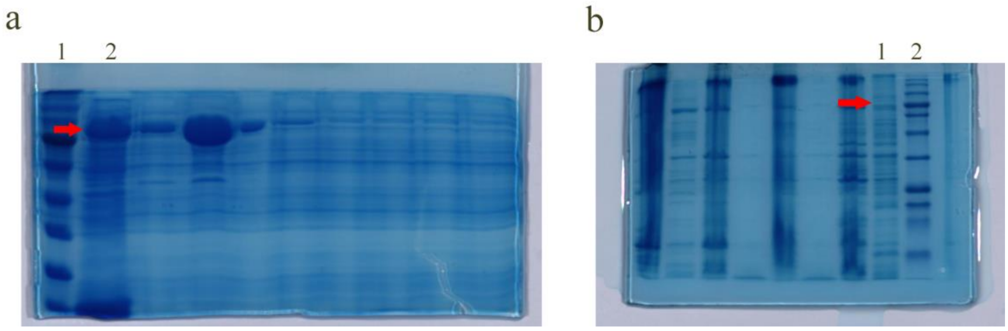

Supplementary Figure S1.The SDS-PAGE original image of **Fig. 3a**. **a**: The electrophoretic results from crude extract of *E. coli* BL-07 that expressed Alg2107. Lane 1 is the Protein Marker, the size of bands (from up to down) is 150, 100, 70, 50, 35, 25, 20, 15 kDa, respectively. Lane 2 is the crude extract of *E. coli* BL07 that expressed Alg2107. **b**: The electrophoretic results from the protein crude extract of negative control expressing empty pET-29a plasmids. Lane 1 is the negative control crude extract, and Lane 2 is the Protein Marker, the size of bands (from up to down) is 180, 140, 100, 72, 60, 45, 35, 25, 20, 15, 10 kDa, respectively. The red arrow indicates the theoretically predicted position of protein Alg2107. The samples derive from the same experiment and the gels were processed in parallel.

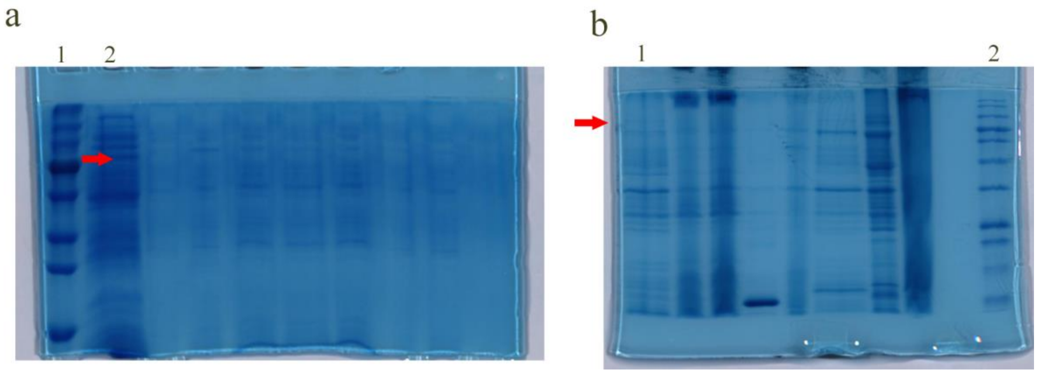

Supplementary Figure S2. The SDS-PAGE original image of **Fig. 3b**. **a**: The electrophoretic results from crude extract of *E. coli* BL-08 that expressed Alg2108. Lane 1 is the Protein Marker, the size of bands (from up to down) is 150, 100, 70, 50, 35, 25, 20, 15 kDa, respectively. Lane 2 is the crude extract of *E. coli* BL08 that expressed Alg2108. **b**: The electrophoretic results from the protein crude extract of negative control expressing empty pET-29a plasmids. Lane 1 is the negative control crude extract, and Lane 2 is the Protein Marker, the size of bands (from up to down) is 180, 140, 100, 72, 60, 45, 35, 25, 20, 15, 10 kDa, respectively. The red arrow indicates the theoretically predicted position of protein Alg2108. The samples derive from the same experiment and the gels were processed in parallel.

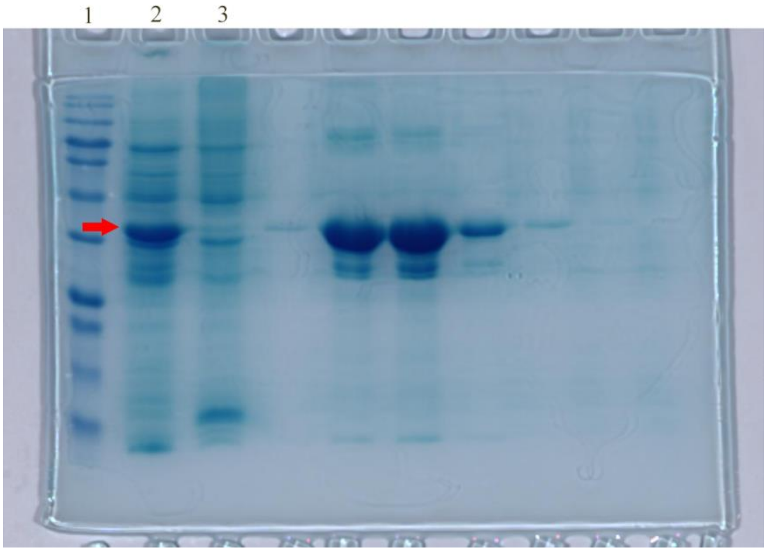

Supplementary Figure S3. The SDS-PAGE original image of **Fig. 3c**. Lane 1 is the Protein Marker, the size of bands (from up to down) is 180, 140, 100, 72, 60, 45, 35, 25, 20, 15, 10 kDa, respectively. Lane 2 is the crude extract of E. coli BL12 that expressed Alg2112. Lane 3 is the protein crude extract of negative control expressing empty pET-29a plasmids. The red arrow indicates the theoretically predicted position of protein Alg2112. The samples derive from the same experiment and gels were processed in parallel.

Supplementary Table S1 Primers of Alginate lyase genes

Primers	Sequences
alg2107-F	GAGCTCATGTCGGCACAAGGTGATTACC
alg2107-R	AAGCTTGGCGTTGTGTGACACCCG
alg2108-F	GGATCCATGGAACAGACATTGCTATTGGATCGCGCAGC
alg2108-R	GTCGACCAGCAAGGCGCACTCGCCTTGCCATT
alg2112-F	GGATCCATGGACGCCGACGGCAATGGCAA
alg2112-R	GTCGACCTGGATCTTGCCGCTTTTCAGC

Supplementary Table S2 genes involved in alginate degradation between *Cobetia* sp. cqz5-12 and *Cobetia Marina* JCM 21022^T.

Source	Gene name or gene cluster	Gene position in genome
Cobetia sp. cqz5-12（This study）	*alg2107*	2493639-2495780
Cobetia sp. cqz5-12（This study）	*alg2108*	2495886-2498045
Cobetia Marina JCM 21022^T [16]	BFX80_RS07330	1725734-1726792
